# Transcriptomic profiling revealed the role of 24-epibrassinolide in alleviating salt stress damage in tall fescue (*Festuca arundinacea*)

**DOI:** 10.3389/fpls.2022.976341

**Published:** 2022-09-22

**Authors:** Yao Chen, Yuanhang Xiang, Zhengrong Hu, Yang Gao, Youxin Zhang, Minghui Chen, A. B. M. Khaldun, Xuebing Yan, Jibiao Fan

**Affiliations:** ^1^College of Animal Science and Technology, Yangzhou University, Yangzhou, China; ^2^Hunan Tobacco Research Institute, Changsha, China; ^3^Bangladesh Agricultural Research Institute, Dhaka, Bangladesh

**Keywords:** 24-epibrassinolide, salt stress, tall fescue, physiological responses, transcriptomic analysis, gene expression

## Abstract

Soil salinization is a major problem all over the world. The accumulation of salt in soil reduces the root water uptake and directly affects plant growth and metabolic activities. Brassinosteroid is a plant hormone that plays an important role in regulation of plant growth and physiological process, including promotion of cell expansion and elongation, signal transduction and stress response. Exogenous 24-epibrassinolide (EBL) has been proved to alleviate various environmental stress in plants. However, the role that EBL plays in salt stress response is still unknown in tall fescue (*Festuca arundinacea*). In this study, the physiology and molecular mechanisms regulated by exogenous EBL of salt stress response in tall fescue was investigated. Tall fescue plants were divided into four groups, including control (CK), NaCl solution (SALT), 24-epibrassinolide (EBL), NaCl solution + 24-epibrassinolide (SE). During the growth period of tall fescue, we found that electrolyte leakage (EL) and malondialdehyde (MDA) were decreased, chlorophyll (Chl) content and antioxidant enzyme activity were increased in leaves of tall fescue in SE group compared with SALT group, indicating that EBL improved the salt tolerance in grasses. Transcriptomic profiling analysis showed that after 12 h of treatments, 10,265, 13,830 and 10,537 differential genes were expressed in EBL, SALT, and SE groups compared with control, respectively. These differentially expressed genes (DEGs) mainly focused on binding, catalytic activity, cellular process, metabolic process, cellular anatomical entity. Moreover, most of the differential genes were expressed in the plant hormone signal transduction pathway. These results helped us to better understand the mechanism of exogenous 24-epibrassinolide to improve the salt tolerance of tall fescue.

## Introduction

Soil salinization is a major problem worldwide. Around 1 billion hectares of land throughout the world are affected by salinity, and these soils are distributed in more than 100 countries (Food and Agriculture Organization of the United Nations, 2015). Soil salinity is induced by natural causes and human behaviors such as improper irrigation (Munns and Tester, 2008). And the accumulation of salt in the soil can seriously affect growth and yield of the plant (Zelm et al., 2020). Soil salinization makes it difficult for roots to absorb water and further disrupt the water potential and ion balance within plant cells. The accumulation of salt in the aerial parts will reduce the photosynthetic capacity of plants and destroy the antioxidant enzyme system, which resulting in a large increase in oxygen free radicals and continuous reduction in growth. And with the extension of time, the damage caused by salt stress is more and more severe, which eventually leads to the stagnation of plant growth or even death (Zhu, 2001; Munns and Tester, 2008; Zelm et al., 2020). The extent and severity of salt-affected lands will worsen due to issues such as global warming (Munns and Gilliham, 2015).

To defense salt stress, plants have developed various strategies to optimize growth conditions under salinity. In addition to regulating plant growth and development, phytohormones also participate in various environmental stresses to improve stress resistance (Yu et al., 2020). Brassinosteroids (BRs) are a new class of plant hormones that are essential for plant growth and development (Kim et al., 2009). Furthermore, it exhibits excellent growth-promoting activity at very low concentrations (Avalbaev et al., 2021). 24-Epibrassinolide (EBL), an active by-product of brassinolide biosynthesis, can stimulate plant metabolic processes such as protein and nucleic acid biosynthesis (Bajguz, 2000). It can significantly improve the ability of plants to resist abiotic stresses, including salt, heavy metal, drought, and low-temperature (Arora et al., 2012; Wu et al., 2017; Anwar et al., 2018; Tanveer et al., 2019).

Tall fescue (*Festuca arundinacea* Schreb) is a broad-leaved outbreeding allohexaploid grass (Hand et al., 2010). As one of the most important cool-season grass species in the world, tall fescue is widely used as turfgrass and forage (Wang et al., 2004). On account of its strong adaptability, tall fescue can grow in temperate and subtropical regions (Cao et al., 2009). Nonetheless, regional salinization limits the growth of tall fescue, which in turn affects turf persistence and forage yield (Wang et al., 2017).

Salinity can cause the increase of reactive oxygen species (ROS) and the content of malondialdehyde (MDA) (Miller et al., 2010; Hu et al., 2012). Plants have a complex antioxidant defense system to alleviate this oxidative damage, including catalase (CAT), peroxidase (POD), superoxide dismutase (SOD) (Lou et al., 2017). In addition, transcriptome analysis is also an available method to study salt stress responses and has been applied to many plants such as Arabidopsis, rice, alfalfa, bermudagrass (Shen et al., 2014; Shi et al., 2015; Wang et al., 2016; Bhattarai et al., 2021). However, there are few studies on the transcriptomic data on the effects of exogenous 24-epibrassinolide in tall fescue under salt stress. Therefore, this study elucidates the mechanism of action of 24-epibrassinolide in alleviating salt stress from both the physiological and transcriptomic levels.

## Materials and methods

### Plant material and growth conditions

Tall fescue (*F. arundinacea*) cultivar Houndog 6 was used for this study and quartz sand was used as growth material. All materials were grown in a climatic chamber (ROX-1000CH, Changzhou, China) at 25°C temperature, 14/10 h (light/dark), 60% relative humidity.

### Treatments

All plants were divided into four groups: control (CK), 1 μmol/L 24-epibrassinolide (EBL), 300 mmol/L NaCl solution (SALT), 300 mmol/L NaCl solution + 1 μmol/L 24-epibrassinolide (SE). Three repetitions were set up for each group, and 100 seeds were evenly sown in each pot. The plants were watered daily until all seeds germinated, then irrigated with 50 ml 1/2 Hoagland nutrient solution per pot. After the plants had grown for 20 days, NaCl and 24-epibrassinolide treatments were conducted. The salt treatment was increased by daily increments of 100 mmol/L NaCl until a final salinity level (300 mmol/L) was reached. Plants were watered with NaCl solution until the liquid flowed from the drain holes at the bottom of the pot. The leaves of plants in the EBL and SE treatments were sprayed with 24-epibrassinolide evenly every day.

### Measurement of chlorophyll content

0.1 g of fresh leaves were immersed in a 15 ml centrifuge tube containing 10 ml of dimethylsulfoxide and were placed in the dark for 48 h. Then, the absorbance of the extraction was measured at 645 and 663 nm with an ultraviolet spectrophotometer (Hiscox and Israelstam, 1979).

Chlorophyll content was calculated with the following formula:


(1) Chltotalcontent(mg⋅L)-1=20.2×OD645+8.02×OD663.

OD645 and OD663 indicate the absorbance of the extraction at 645 and 663 nm, respectively.

### Measurement of electrolyte leakage

The determination of electrolyte leakage (EL) referred to the method of Feng et al. (2021) with slight modifications. In detail, 0.1 g of fresh leaves were chopped and placed in a 50 ml centrifuge tube containing 25 ml of deionized water. The tubes were shaken at room temperature for 24 h at 200 rpm. The initial conductivity (Ci) was measured with a conductivity meter (DDS-11A conductivity Meter, Hangzhou, China). Then, the leaf tissue in the tube was autoclaved at 121°C for 20 min to release the electrolytes completely. The conductivity (Cmax) was measured again after the solution had been cooled to room temperature. The EL was calculated by the formula:


$$\mathrm{EL}\left(\%\right)=\frac{\text{Ci}}{\text{Cmax}}\times 100\%$$

### Crude enzyme extraction

0.2 g of fresh leaves were quickly ground into powder with liquid nitrogen. 4 mL of pre-chilled sodium phosphate buffer (pH 7.8) was added to the powder and the homogenate was transferred to a 15 mL centrifuge tube. After centrifugation at 4°C at 12,000 rpm for 15 min, the supernatant was extracted as crude enzyme solution.

### Measurement of malonaldehyde content

Malonaldehyde (MDA) content was determined by thiobarbituric acid (TBA) method (Fan et al., 2015). Add 1 ml of crude enzyme solution to 2 ml of MDA reaction buffer containing 0.5% (v/v) TBA and 20% (v/v) trichloroacetic acid. The mixed solution was water-bathed at 95°C for 30 min and then rapidly cooled to room temperature in ice. Then the tubes were centrifuged for 10 min at 25°C with 12,000 rpm. The solution was measured at 532 and 600 nm with an ultraviolet spectrophotometer. MDA content was calculated with following formula:


MDA⁢(molg⁢FW-1)=[(OD532-OD600)×L](l×ε×FW).

Where L is the volume of the extraction, l is the thickness of the cuvette, ε is the molar absorption coefficient of 155 mM^–1^ cm^–1^, and FW is the fresh weight of the leaves.

### Quantification of antioxidant enzymes

To determine CAT activity, 0.1 ml of crude enzyme solution was added to a mixed solution containing 2 ml phosphate buffer (100 mM, pH 7.0), 0.5 ml 100 mM hydrogen peroxide (H_2_O_2_). Absorbance at 240 nm was measured three times with an ultraviolet spectrophotometer and the values were recorded per minute. A reduction of one unit of the absorbance per minute was defined as one unit CAT activity.

To determine POD activity, 20 μL of crude enzyme solution was added to a mixed solution containing 2.5 mL phosphate buffer (100 mM, pH 6.0), 1.4 μL guaiacol, 0.95 μL 30% hydrogen peroxide (30% H_2_O_2_). Absorbance at 470 nm was measured three times with an ultraviolet spectrophotometer and the values were recorded per minute. Increment of one unit of the absorbance per minute was defined as one unit POD activity.

To determine SOD activity, 0.1 mL of crude enzyme solution was added to a mixed solution containing 1.5 ml phosphate buffer (50 mM, pH 7.8), 0.3 ml 130 mM methionine (Met), 0.3 ml 750 μM nitroblue tetrazolium (NBT), 0.3 ml 100 μM disodium ethylenediamine tetraacetic acid (EDTA-Na_2_), and 0.3 mL 20 μM riboflavin. Take the mixed solution without crude enzyme solution as control. Another new tube was regarded as blank, in which phosphate buffer (50 mM, pH 7.8) was used instead of the crude enzyme solution. The control and the experimental groups were placed under the condition of 4,000 Lux for 30 min, and the blank was placed in the dark for 30 min. Absorbance at 560 nm was measured with an ultraviolet spectrophotometer and the blank was used as zero adjustment. Taking NBT photoreduction by 50% as one unit of SOD activity.

### RNA extraction and library construction

Total RNA was extracted from plant tissues using the ethanol precipitation protocol and CTAB-PBIOZOL reagent according to the manufacturer’s instructions. The concentration and purity of RNA were tested by the Agilent 2100 Bioanalyzer (Thermo Fisher Scientific, MA, USA) and NanoDrop. Use DNase I to digest the DNA fragments presenting in total RNA samples. The magnetic beads were purified to recover the reaction products. RNase H (Illumina, CA, USA) was used to remove the rRNA. Purified mRNA from previous steps was fragmented into small pieces with fragment buffer at appropriate temperature. Then, First-strand cDNA was generated in First Strand Reaction System by PCR, and the second-strand cDNA was generated as well. The reaction product was purified by magnetic beads. A-Tailing Mix and RNA Index Adapters were added by incubating to carry out end repair. The cDNA fragments with adapters were amplified by PCR. The PCR products were heat denatured into single-stranded DNA, and a single-stranded circular DNA library was obtained by circularizing the single-stranded DNA with a bridge primer.

### Sequencing data filtering and *de novo* assembly

Raw reads were filtered via SOAPnuke (v1.4.0) (Chen et al., 2018) by removing reads containing adaptors, poly-N or low quality, and then clean reads were obtained. Trinity (v2.0.6) (Grabherr et al., 2011) was used to assemble the clean reads and Tgicl (v2.0.6) (Pertea et al., 2003) to perform clustering and eliminate redundant data in the assembled transcripts to obtain unique genes. Unigenes were divided into two parts. One part were clusters, which were the results of further de-redundancy. And there were several unigenes with high similarity (greater than 70%) in the same cluster, starting with CL, followed by the number of the gene family. The rest were singletons, starting with Unigene, referring to a single unigene without clustering.

### Coding DNA sequence forecast and unigene annotation

The candidate coding regions in Unigene were identified using TransDecoder (v3.0.1) (Kim et al., 2015), the longest open reading frame was extracted. Based on sequence similarity, Diamond Blastp was used to align to SwissProt database, and then Hmmscan (v3.0) was used to screen the Blast results in the Pfam database. Trans Decoder. Predict was used to predict coding regions finally. The assembled unigenes were annotated with seven functional databases (KEGG, GO, NR, NT, SwissProt, Pfam, and KOG).

### Gene expression analysis

Gene expression levels in each sample were calculated by RSEM (v1.2.8) (Li and Dewey, 2011) and expression analysis of differential genes was performed by the DESeq2 with Qvalue (adjusted *p*-value) ≤ 0.05 (Anders and Huber, 2010; Love et al., 2014).

### Quantitative real-time PCR

cDNA was synthesized using the HiScript^®^ III RT SuperMix for qPCR (+ gDNA wiper) (Vazyme, Nanjing, China). Quantitative real-time PCR (qRT-pCR) reactions were performed on QuantStudio™ 3 Real-Time PCR System (Thermo Fisher Scientific, MA, USA) using the AceQ^®^ Universal SYBR qPCR Master Mix (Vazyme, Nanjing, China). The thermal cycle program was as following: 95°C for 5 min, 40 cycles of 95°C for 10 s and 60°C for 30 s; 95°C for 15 s, 60°C for 1 min, 95°C for 15 s. The information of primers for the genes for qRT-PCR are listed in Supplementary Table 1. The expression data were calculated by the 2^–ΔΔ*Ct*^ method (Pfaffl, 2001).

## Results

### Effects of exogenous 24-epibrassinolide on growth and physiological responses under salt stress

**FIGURE 1:**
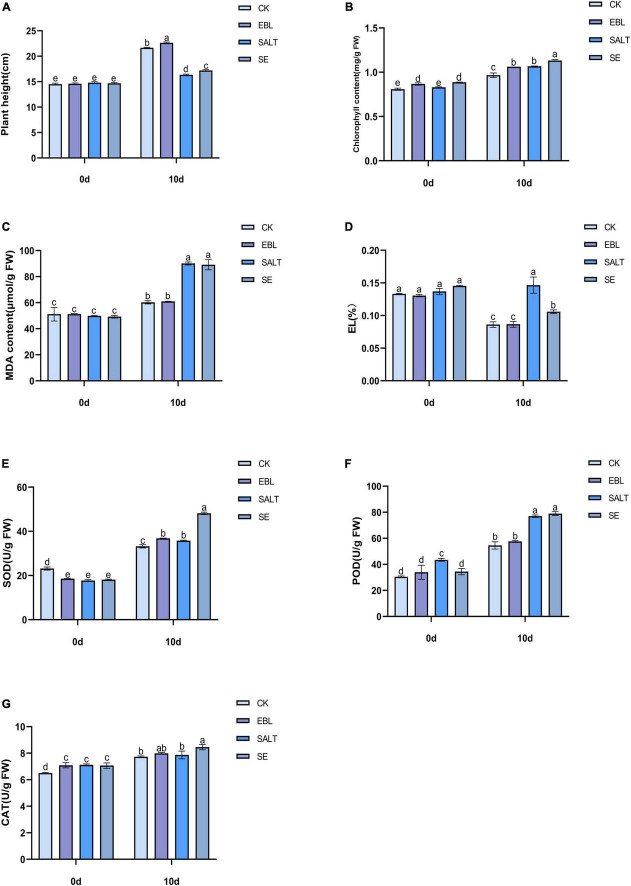
Effects of exogenous 24-epibrassinolide on growth and physiological characteristics of tall fescue under salt stress. **(A)** Plant height of tall fescue; **(B)** Chlorophyll content of tall fescue; **(C)** MDA content; **(D)** Electrolyte Leakage; **(E)** SOD activity; **(F)** POD activity; **(G)** CAT activity. Bars with different letters indicate significant difference at *P* ≤ 0.05 (Duncan test).

In order to investigate whether exogenous 24-epibrassinolide could improve the growth of tall fescue under salt stress, the plant height was measured. The results showed that the plant height of the four groups increased at 10 days after different treatments. Among them, the two groups (SALT and SE) treated with NaCl solution were significantly lower than the CK and the EBL. After application of 24-epibrassinolide, the plant heights of EBL and SE groups were increased by 4.5% and 5.2% compared with the CK and SALT, respectively (Figure 1A and Supplementary Figure 1). These results indicated that 24-epibrassinolide can enhance the growth of tall fescue under salt stress remarkably.

To investigate the effect of 24-epibrassinolide on the chlorophyll of the leaves under salt stress, the chlorophyll content was determined. After 10 days of treatment, the chlorophyll content in the leaves after SE treatment was the highest, followed by SALT and EBL, and the content of CK was the lowest. With the application of 24-epibrassinolide, the chlorophyll content in the leaves after EBL and SE treatments increased by 9.9% and 6.1%, respectively compared with CK and SALT (Figure 1B). These results implied that exogenous 24-epibrassinolide could maintain the chlorophyll stability of tall fescue under salt stress.

To investigate whether the exogenous 24-epibrassinolide played a positive role in maintaining cell membrane stability of tall fescue under salt stress, MDA content and EL alterations were determined. The contents of MDA and EL in tall fescue after 10 days of salt treatment were much higher than those of the CK. However, there were no significant differences observed in MDA and EL contents between CK and EBL groups. After exogenous application of 24-epibrassinolide, MDA and EL contents of SE treatment decreased by 1.09% and 27.8% compared with the SALT, respectively (Figures 1C,D). These results suggested that exogenous 24-epibrassinolide could maintain cell membrane stability.

To investigate the effects of 24-epibrassinolide on antioxidant enzymes, activities of SOD, POD and CAT were determined (Figures 1E–G). After 10 days of salt treatment, the activities of SOD and POD were enhanced, but CAT had no significant difference compared with control. After the application of 24-epibrassinolide, activities of SOD, POD and CAT increased by 10.7, 5.5, and 3.4% in the leaves of EBL treatment compared with the CK. The SOD, POD, and CAT activities in plants of SE treatment were 34.6, 2.5, and 7.5% higher than those of SALT, respectively. These results demonstrated that 24-epibrassinolide plays an important role in enhancing antioxidative enzyme activities of tall fescue in response to salt stress.

### Transcriptome sequencing analysis and unigene annotation

**FIGURE 2:**
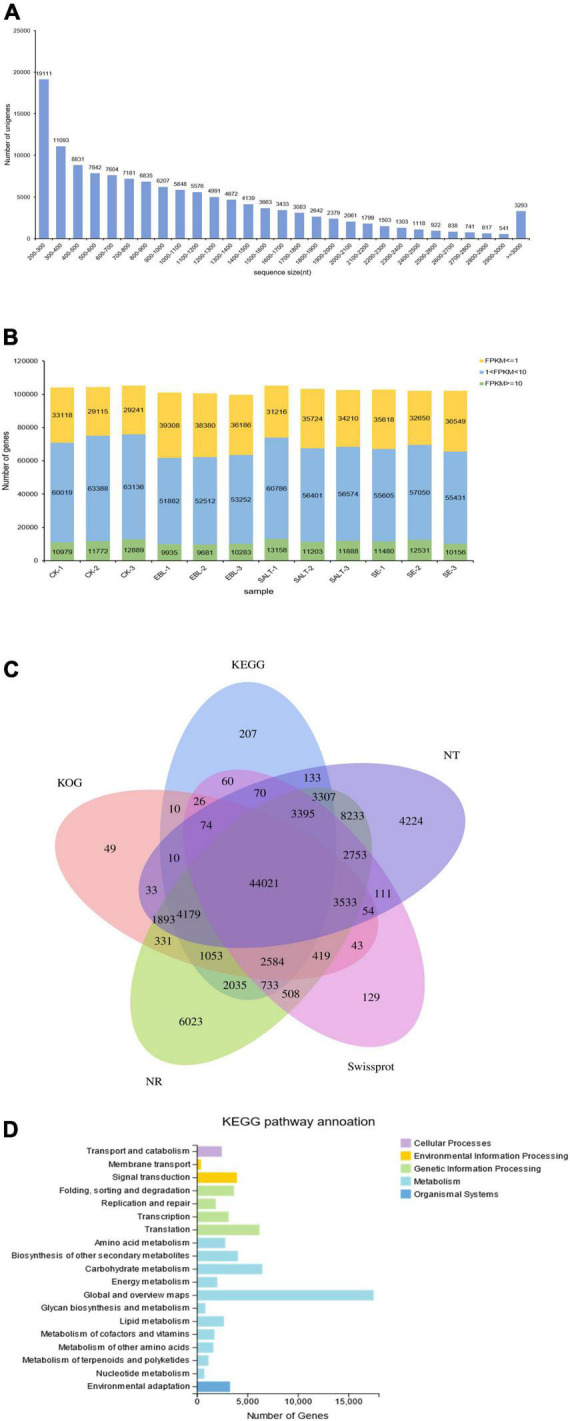
Overview of the tall fescue transcriptome dataset. **(A)** Unigene length distribution; **(B)** Number of genes in different FPKM intervals for each sample; **(C)** Venn diagram of KEGG, NT, NR, KOG, and Swissprot annotation results of the bermudagrass reference transcriptome; **(D)** Distribution and quantity statistics of KEGG functions.

The transcriptome sequencing obtained 129,866 unigenes after *de novo* assembly and de-redundancy. The total length, average length, N50 and GC content were 135,692,978 bp, 1,044 bp, 1,466 bp, and 48.89%, respectively (Supplementary Table 2). Among them, 19,111 unigenes with a length of 200–300 bp accounted for the largest number, and the number of unigenes with a length of 2,900-3,000 bp accounted for the least, with a total of 541. It is worth noting that there were 3,293 genes with length greater than or equal to 3,000 bp (Figure 2A). It can be seen from the expression stacking chart that the Fragments Per Kilobase of exon model per Million mapped fragments (FPKM) values between 1 and 10 in each group accounted for the most, and the number greater than 10 was the least (Figure 2B).

Unigenes were compared to seven functional databases for annotation, there were 85,000 in NR (65.45%), 76,023 in NT (58.54%), 58,513 in SwissProt (45.06%), 58,312 in KOG (44.90%), 61,897 in KEGG (47.66%), 61,289 in GO (47.19%) and 58,434 in Pfam (45.00%) (Figure 2C and Supplementary Table 3). We used the KEGG database to analyze pathway enrichment for all unigenes. As a result, 61,897 unigenes were annotated into five categories: cellular processes, environmental information processing, genetic information processing, metabolism, and organismal system. In this classification, genes were further divided into 19 functional pathways. Metabolic categories had the most types of enriched pathways, among which, carbohydrate metabolism accounts for the largest proportion except the global and overview maps. Genetic information processing was the second functional category with four subcategories. Although cellular processes and environmental information processing both have only one subclass, 2,479 and 3,288 single genes were enriched respectively (Figure 2D). In addition, the transcription factor families in which the unigenes belong were classified and counted. The results showed that the most genes belonged to the MYB family, followed by the bHLH family (Supplementary Table 4).

### Identification and gene ontology analysis of differentially expressed genes

**FIGURE 3:**
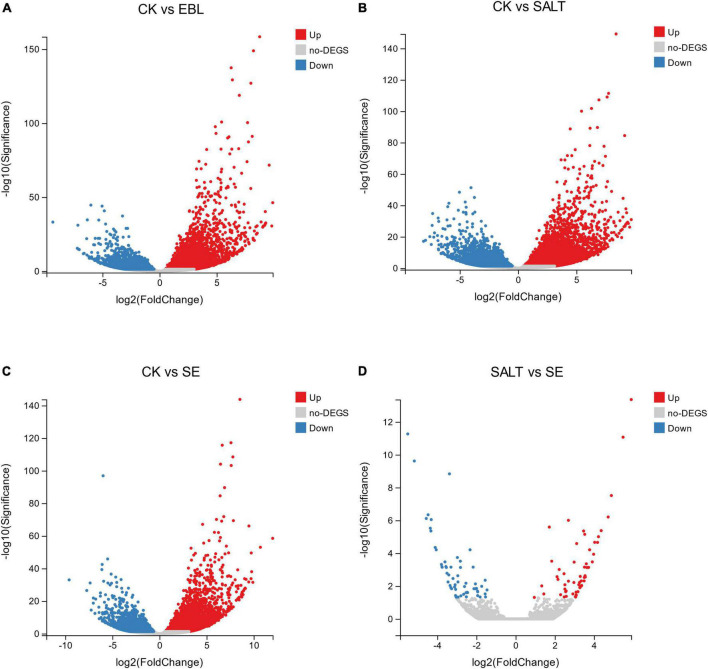
Differential genes between groups in volcano maps. **(A)** The distribution of differential genes between CK and EBL; **(B)** The distribution of differential genes between CK and SALT; **(C)** The distribution of differential genes between CK and SE; **(D)** The distribution of differential genes between SALT and SE. Up-regulated genes were shown in red and down-regulated genes were shown in blue.

We performed volcano plots for the differential gene expression of the four comparison groups. Compared with CK, EBL group had 6,618 genes up-regulated and 3,647 genes down-regulated, respectively. In the CK vs. SALT comparison, there were 13,830 different genes, including 7,968 up-regulated genes and 5,862 down-regulated genes. There were 10,537 differential genes in the groups of CK and SE, including 6,409 up-regulated genes and 4,128 down-regulated genes. In the SALT vs. SE comparison, 117 DEGs were obtained, containing 58 upregulated genes and 59 downregulated genes (Figure 3).

**FIGURE 4:**
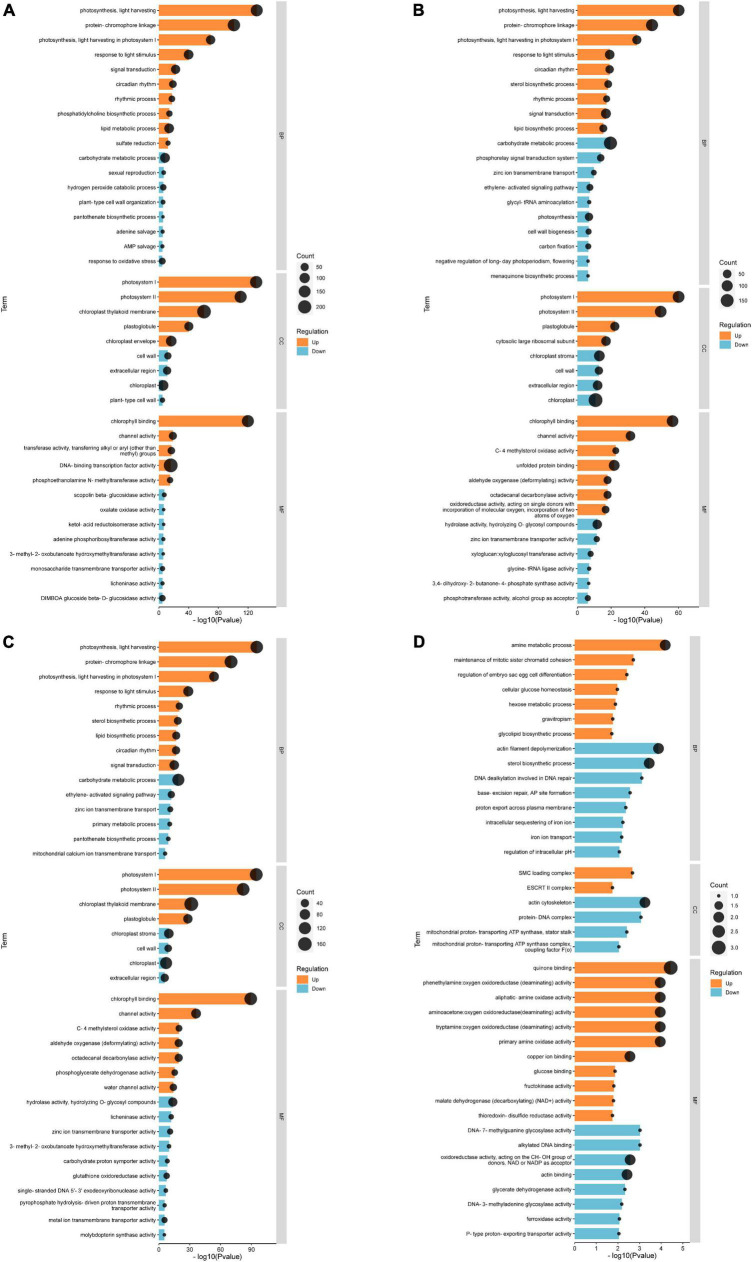
Gene ontology (GO) classification of genes with significant differences between groups. **(A)** GO classification of differential genes between CK and EBL; **(B)** GO classification of differential genes between CK and SALT; **(C)** GO classification of differential genes between CK and SE; **(D)** GO classification of differential genes between SALT and SE. The top 20 up-regulated genes between two groups were shown in orange and the top 20 down-regulated genes were shown in blue.

Based on the results, we selected the top 20 up-regulated and 20 down-regulated genes with the smallest Q-value in each comparison group for Gene Ontology (GO) analysis. According to GO annotation, differentially expressed genes (DEGs) are divided into three functional categories: molecular function (MF), cellular component (CC), and biological process (BP). And each functional category contains multiple levels of subcategories. In the CK vs. EBL comparison and the CK vs. SALT comparison, the most enriched GO term among the up-regulated genes were DNA-binding transcription factor activity in MF and protein-chromophore linkage in BP, respectively. The term with the highest number of down-regulated genes for both comparison groups was chloroplast in CC (Figures 4A,B). In the CK vs. SE comparison, chloroplast thylakoid membrane in CC was the most in up-regulated genes. Among down-regulated genes, carbohydrate metabolic process in BP and chloroplast in CC occupied the most by the same count (Figure 4C). In the SALT vs. SE comparison, the most abundant term in up-regulated genes was quinone binding in MF. The terms in down-regulated genes were actin filament depolymerization, sterol biosynthetic process in BP, actin cytoskeleton in CC, actin binding and oxidoreductase activity, acting on the CH-OH group of donors, NAD or NADP as acceptor in MF, respectively (Figure 4D).

### Trend analysis of differentially expressed genes among different comparisons

**FIGURE 5:**
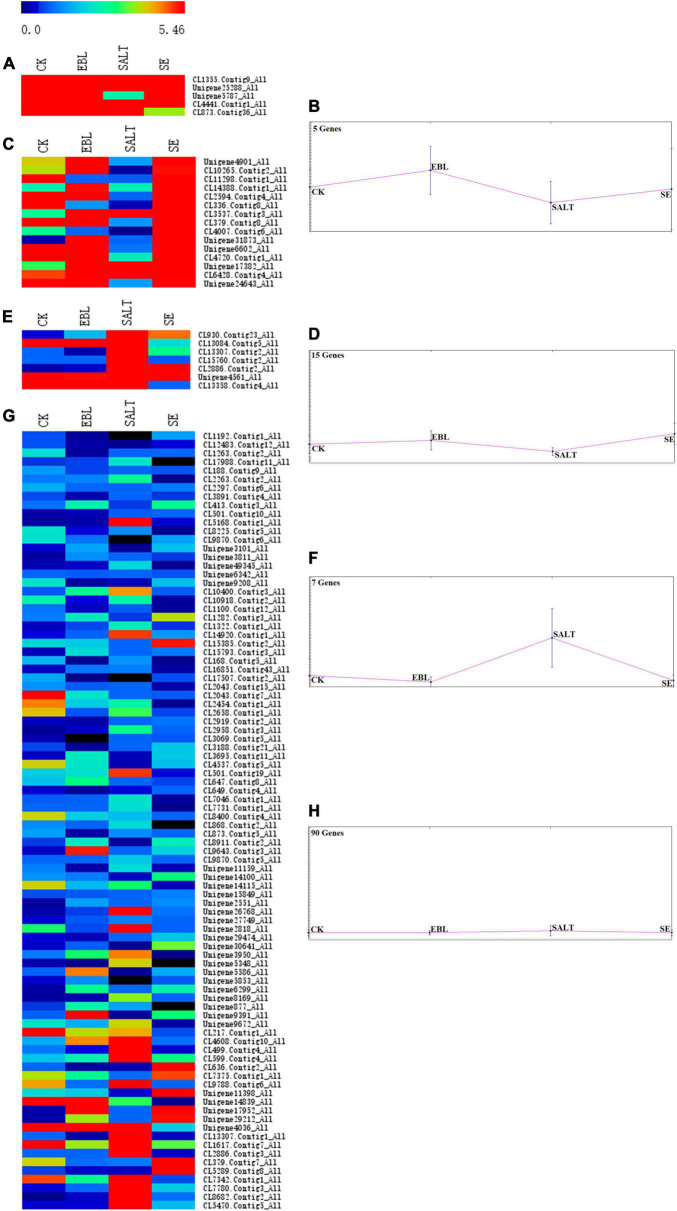
Trend analysis of DEG expression in four groups. **(A)** Heatmap of gene expression in cluster 1; **(B)** Trend graph of gene expression in cluster 1; **(C)** Heatmap of gene expression in cluster 2; **(D)** Trend graph of gene expression in cluster 2; **(E)** Heatmap of gene expression in cluster 3; **(F)** Trend graph of gene expression in cluster 3; **(G)** Heatmap of gene expression in cluster 4; **(H)** Trend graph of gene expression in cluster 4.

A bidirectional clustering heatmap was created based on the 117 DEGs of SALT vs. SE comparison. DEGs with similar expression patterns were divided into multiple clusters using the k-means clustering method. Each cluster contained a heatmap and a trend graph. There were CL1355.Contig9, Unigene25288, Unigene5787, CL4441.Contig1, CL873.Contig36 in cluster 1 and the expression trends in the four groups were increased, and then decreased and finally increased again. There were 15 genes in cluster 2. Its trend was the same as cluster 1, but the fluctuation was smoother. Seven genes were in cluster 3 and the expression level of SALT was the highest among the four groups. There were 90 genes in cluster 4. It had the lowest gene expression among the four clusters, and the fluctuation of its trend was the smallest (Figure 5 and Supplementary Figure 2).

### Plant hormone signal transduction

**FIGURE 6:**
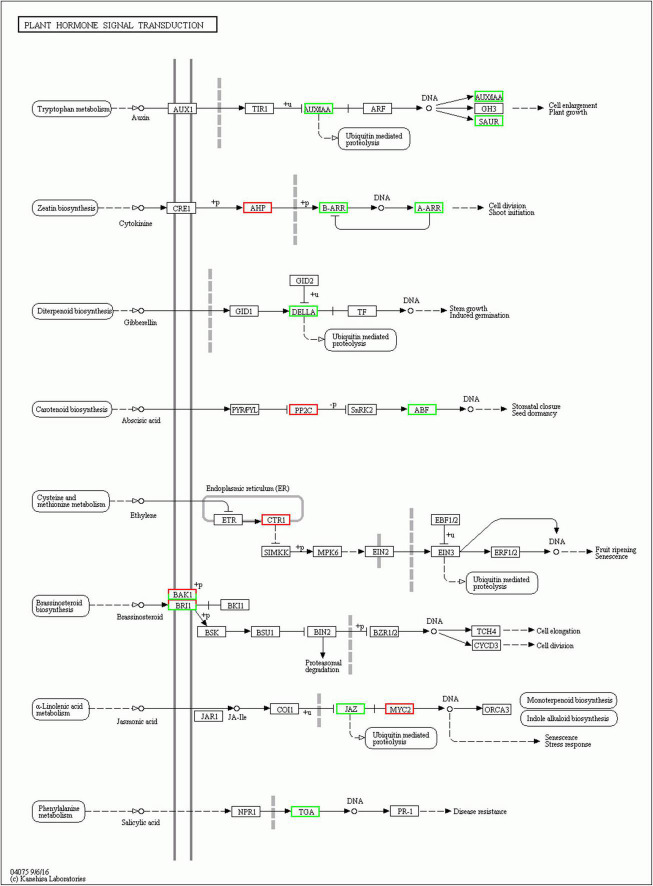
Plant hormone signal transduction in EBL vs. SE comparison. Up-regulated DEGs were indicated in red, down-regulated DEGs were indicated in green.

Plant hormones play important roles in growth and development of plants. Here, genes involved in plant hormone signal transduction pathways were differentially expressed in EBL vs. SE comparison (Figure 6). Auxin is an important regulatory hormone in plants. Several components of auxin signal transduction pathway were down-regulated after SE treatment compared to EBL, including one auxin-responsive protein IAA (Aux/IAA), one SAUR family protein (SAUR). There were three DELLA proteins (DELLA) down-regulated in the gibberellin pathway. Similarly, the salicylic acid pathway involved in disease resistance also contains only down-regulated genes. There was an up-regulated Arabidopsis histidine-containing phosphotransfer protein (AHP), two down-regulated genes of two-component response regulator ARR-B family (B-ARR) and two-component response regulator ARR-A family (A-ARR) in the cytokinin transduction pathway. In the ethylene transduction pathway related to fruit ripening and senescence, genes were all up-regulated. Most genes were also up-regulated in the abscisic acid signaling pathway. For jasmonic acid, three jasmonate ZIM domain-containing protein (JAZ) were down-regulated and three transcription factor MYC2 (MYC2) were up-regulated after SE treatment. In the brassinosteroid pathway, both brassinosteroid insensitive 1-associated receptor kinase 1 (BAK1) and protein brassinosteroid insensitive 1 (BRI1) contain one up-regulated and one down-regulated gene, respectively. Compared with SALT, SE had up-regulated genes in auxin and cytokinin signal transduction pathways, which indicated that the application of 24-epibrassinolide could improve plant growth (Supplementary Figure 3).

### Verification of differentially expressed genes

**FIGURE 7:**
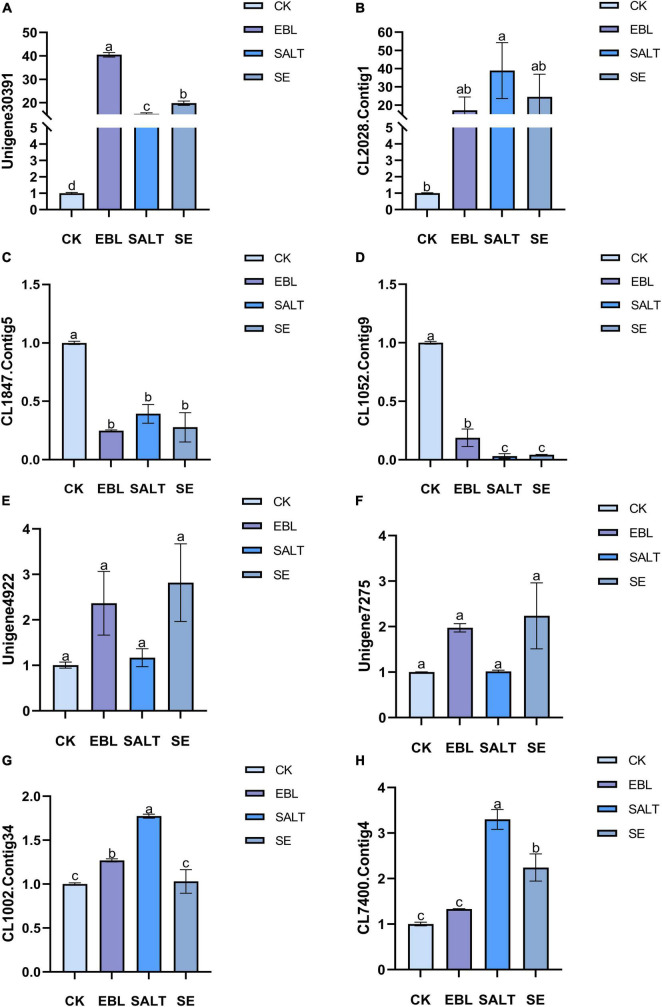
Quantitative real-time PCR (qRT-pCR) analysis of several differentially expressed genes in tall fescue. **(A)** Relative expression of Unigene30391; **(B)** Relative expression of CL2028.Contig1; **(C)** Relative expression of CL1847.Contig5; **(D)** Relative expression of CL1052.Contig9; **(E)** Relative expression of Unigene4922; **(F)** Relative expression of Unigene7275; **(G)** Relative expression of CL1002.Contig34; **(H)** Relative expression of CL7400.Contig4. Bars with different letters indicate significant difference at *P* ≤ 0.05 (Duncan test).

We selected eight DEGs for transcriptome sequencing validation (Figure 7 and Supplementary Figure 4). Two genes were up-regulated in the three treated groups compared to the control group, and two genes were down-regulated in the three treated groups compared with the control group. Among the remaining four genes, two were expressed in the EBL and the SE, and the other two were expressed in the SALT or the SE. The expression levels of Unigene30391 and CL2028.Contig1 in the three treatment groups were higher than those of CK, and the expression levels of these two genes were the highest in EBL and SALT groups, respectively. CL1847.Contig5 and CL1052.Contig9 were both down-regulated in the three treatment groups, and the expression levels of these two genes were the lowest in EBL and SALT groups, respectively. Unigene4922 and Unigene7275 were only expressed in EBL and SE groups, and were significantly higher than CK and SALT. The expression of CL1002.Contig34 was the largest in SALT group, SE was the same as CK, and EBL was slightly larger than CK. CL7400.Contig4 was expressed in both SALT and SE, and their expression levels were two times and one time higher than that of CK, respectively.

## Discussion

In saline environments, plants develop multiple strategies at the physiological and molecular levels to mitigate the damage caused by salt stress. The results of this study showed that NaCl treatment inhibited the growth and physiology of tall fescue, while the addition of EBL could confer increased tolerance to salt stress. Under the influence of sodium chloride, the plant height and chlorophyll content of tall fescue decreased, while the MDA content and electrolyte leakage increased. These growth and physiological changes were improved when 24-epibrassinolide was used. We found that SOD and CAT activities of tall fescue increased under salt stress, which was consistent with the results of Xu et al. (2013). After adding EBL, the activity of the antioxidant enzyme system was enhanced to improve the resistance of plants to salt stress. Wu et al. (2017) also found that exogenous 24-epibrassinolide increased the chlorophyll content of perennial ryegrass, enhanced the activities of SOD and CAT, and decreased the EL and MDA content. Similarly, Alam et al. (2019) found that EBL reduced the electrolyte leakage and hydrogen peroxide content, as well as the accumulation of Na^+^ of soybean. Moreover, Kolomeichuk et al. (2020) demonstrated that 24-epibrassinolide could increase peroxidase activity and improve the K^+^/Na^+^ ratio in potato leaves under NaCl treatment. These results suggest that 24-epibrassinolide may be an effective molecule for enhancing plant salt tolerance.

In order to understand the response mechanism of 24-epibrassinolide in alleviating salt stress, DEGs were subjected to GO analysis. In the CK vs. SALT comparison (Supplementary Table 5), most DEGs were annotated to cellular anatomical entity (6,045) in cellular component, catalytic activity (4,429) and binding (4,423) in molecular function, cellular process (3,990) and metabolic process (3,613) in biological process. The results were accorded with the GO annotation results of differential genes in tall fescue after 24 h of salt treatment by Amombo et al. (2018). Interestingly, the top five annotation categories in the CK vs. EBL comparison (Supplementary Table 5) were the same as CK vs. SALT. We randomly selected several genes for validation, and these genes were up-regulated or down-regulated under salt or 24-epibrassinolide treatment compared to the control. The GO annotations of these genes in the molecular function were calcium ion binding, chlorophyll binding, DNA binding and ATP binding, respectively. In addition to salt stress, most of the molecular functional subcategories of GO analysis are binding under drought, heat and lead stress (Li et al., 2017; Shu et al., 2020; Liu et al., 2021; Ouertani et al., 2021). This suggests that binding-related genes play a significant role in plant resistance to various environmental stresses.

There are various signals and pathways in plants to enhance salt tolerance (Yang and Guo, 2018; Zelm et al., 2020). Salt stress induces ionic and osmotic stress, leading to an increase in cytoplasmic Ca^2+^ content, and then the SOS pathway is triggered to export excess Na^+^ to reduce salt toxicity (Zhao et al., 2021). In addition, the high-affinity potassium transporter (HKT1), the mitogen-activated protein kinases (MAPKs) and various plant hormones are also important anti-stress signals (Pan et al., 2011; Fujita et al., 2013; Zhang et al., 2013; Khan et al., 2019; Nolan et al., 2020; Zhou et al., 2020). In this study, the plant hormone signal transduction, MAPK signaling pathway-plant and plant-pathogen interaction were the most enriched signal transduction pathways in the signal transduction of DEGs. Salt stress is regulated by the interaction of multiple plant hormones, and auxin is a key mediator in this process (Ribba et al., 2020). Auxin is perceived by Transport Inhibitor Response 1 (TIR1), which ubiquitinates Aux/IAA. Then the inhibition of AUXIN RESPONSE FACTORS (ARFs) is released, and auxin-induced genes expression are activated (Leyser, 2018). The results showed that genes related with auxin were down-regulated after SE treatment compared to EBL. In addition, among the other three hormones related to plant growth, gibberellin genes were all down-regulated, most genes of cytokinins and half of brassinosteroids were also down-regulated. However, there were up-regulated genes in the ethylene and jasmonic acid pathways associated with senescence. Growth-related genes were reduced, while defense-related genes were increased. This may be a protective mechanism adopted by plants to resist the damage caused by external stress (He et al., 2022).

Most transcription factors such as MYB, WRKY, bHLH, and NAC play important roles in plant adaptation to salt stress (Baillo et al., 2019). Many MYB proteins have been implicated in salt tolerance. MYB74 and MYB20 can be induced and the interaction between MPK4 and MYB42 can be enhanced by NaCl treatment (Wang et al., 2021). Among the transcription factor families related to plant stress resistance, the bHLH family is the second largest family after the MYB family. bHLH is an indispensable factor in the salt tolerance of various plants (Sun et al., 2018). The top six transcription factors that we annotated in the TF database with the highest number were MYB, bHLH, AP2-EREBP, WRKY, FAR1, NAC. Moreover, the genes annotated to these six families were both up-regulated and down-regulated after NaCl and 24-epibrassinolide treatment, indicating that these transcription factors were actively involved in the salt response of tall fescue.

## Conclusion

To better understand the effect of 24-epibrassinolide on the salt tolerance of tall fescue, we performed studies at the physiological and transcriptomic levels. Through RNA sequencing, a total of 129,866 unigenes were acquired in four groups of CK, EBL, SALT, and SE. These unigenes were annotated in seven functional databases. A large number of DEGs were obtained in different comparison groups. There were 10,265 and 13,830 DEGs in EBL and NaCl treatments compared with control, respectively. Most of them were annotated in metabolic process, binding, catalytic activity in GO database. Most of the DEGs were involved in plant signal transduction pathways, indicating that hormones actively mediated the salt response mechanism of tall fescue. At the physiological level, the plant height of tall fescue was significantly decreased and the level of membrane lipid peroxidation was increased under NaCl treatment. After the application of 24-epibrassinolide, the contents of MDA and EL in leaves were remarkably reduced, and the content of chlorophyll and the activities of antioxidant enzymes were enhanced. These results suggest that 24-epibrassinolide has non-negligible effects on the enhancement of plant salt tolerance.

## Data availability statement

The data presented in the study are deposited in the China National Center repository, accession number PRJCA010198 (https://ngdc.cncb.ac.cn/bioproject/browse/PRJCA010198).

## Author contributions

YC, JF, XY, and ZH conceived and designed the research. YC, YX, YG, YZ, and MC performed the experiments. YC analyzed the data and drafted the manuscript. YC, JF, and AK revised the manuscript. All authors contributed to the article and approved the submitted version.

## Supplementary material

Supplementary Figure 1 Growth of tall fescue after 10 days of 24-epibrassinolide and salt stress treatments.

Supplementary Figure 2 A bidirectional clustering heatmap of DEGs in the SALT vs. SE comparison.

Supplementary Figure 3 Plant hormone signal transduction in SALT vs. SE comparison.

Supplementary Figure 4 FPKM values of genes used for qRT-PCR analysis. (A) FPKM values of Unigene30391; (B) FPKM values of CL2028.Contig1; (C) FPKM values of CL1847.Contig5; (D) FPKM values of CL1052.Contig9; (E) FPKM values of Unigene4922; (F) FPKM values of Unigene7275; (G) FPKM values of CL1002.Contig34; (H) FPKM values of CL7400.Contig4.
