# Supplementary figures and images for: Transcriptomic profiling revealed the role of 24-epibrassinolide in alleviating salt stress damage in tall fescue (Festuca arundinacea)
Source: Front Plant Sci. 2022 Sep 22;13:976341. doi: 10.3389/fpls.2022.976341 (PMC9540362; doi:10.3389/fpls.2022.976341)

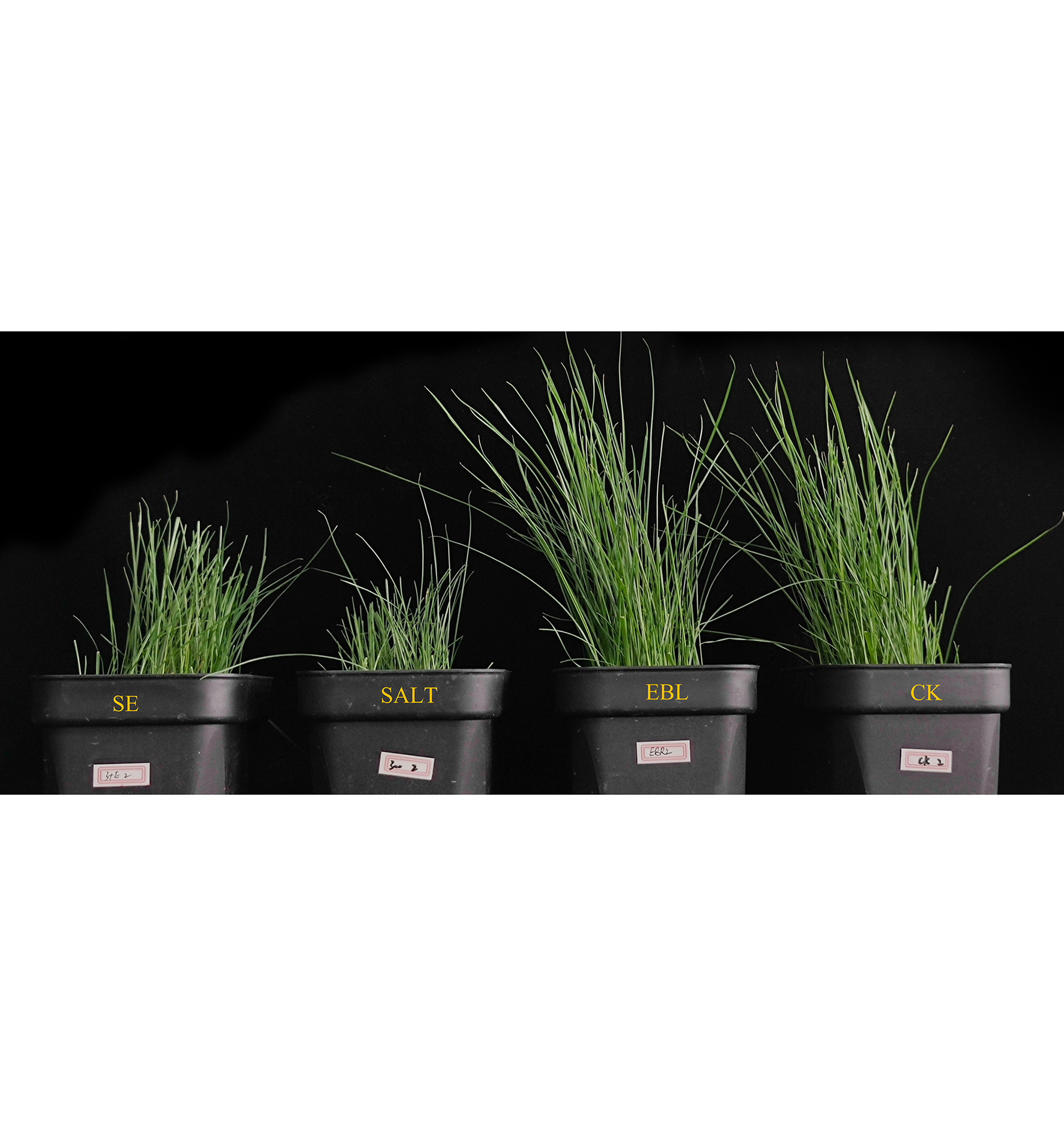

Supplement: Supplementary Figure 1 — Growth of tall fescue after 10 days of 24-epibrassinolide and salt stress treatments. [file Image_1.TIF]

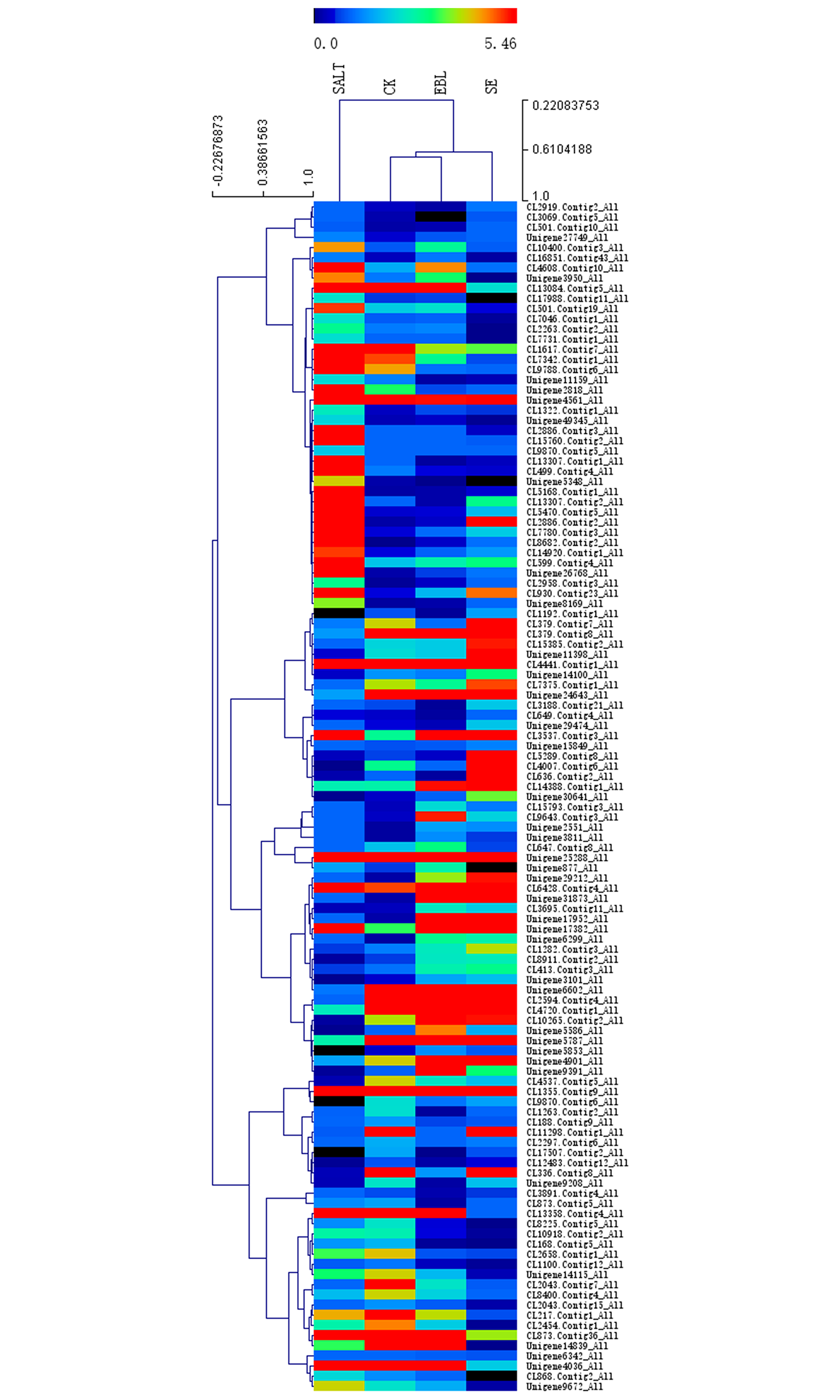

Supplement: Supplementary Figure 2 — A bidirectional clustering heatmap of DEGs in the SALT vs. SE comparison. [file Image_2.TIF]

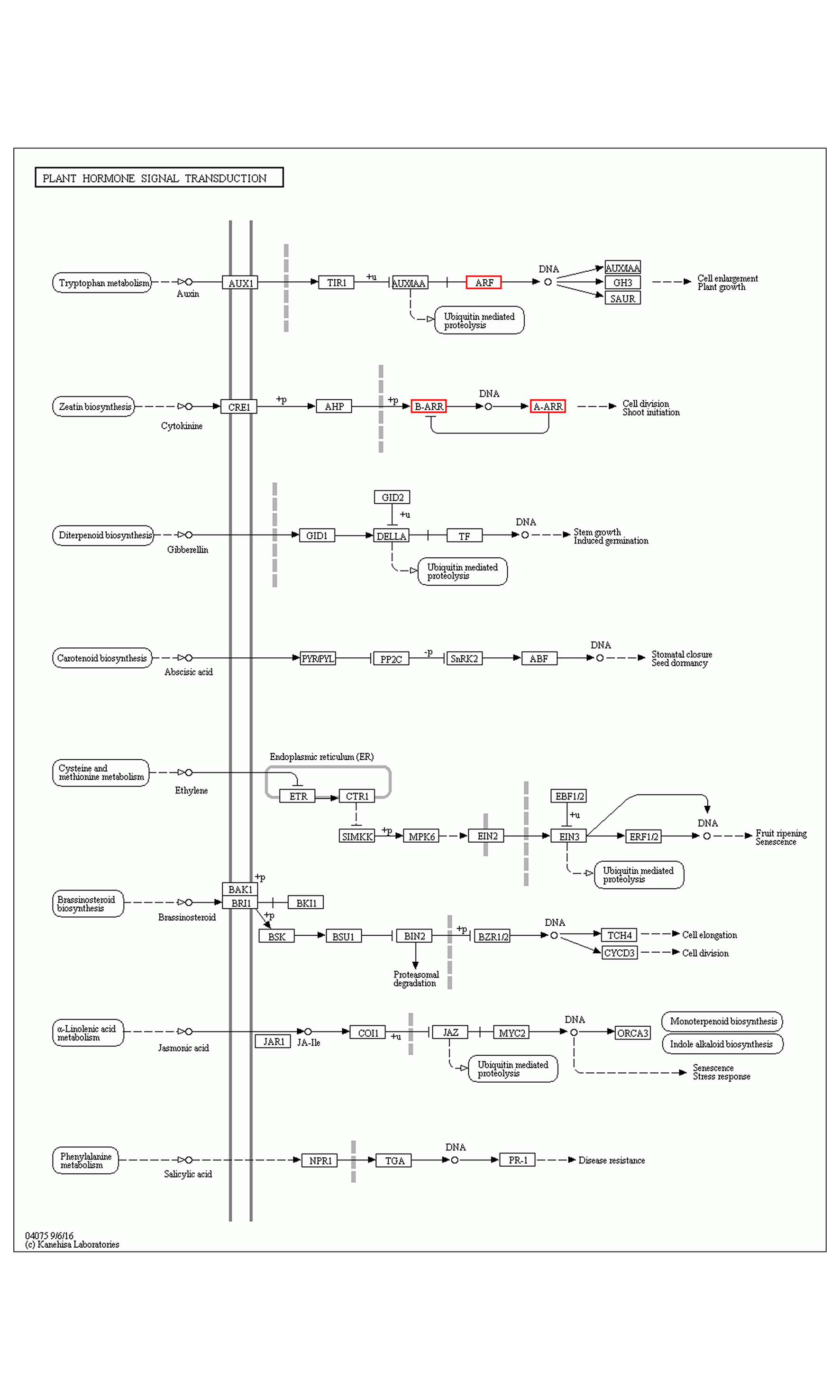

Supplement: Supplementary Figure 3 — Plant hormone signal transduction in SALT vs. SE comparison. [file Image_3.TIF]

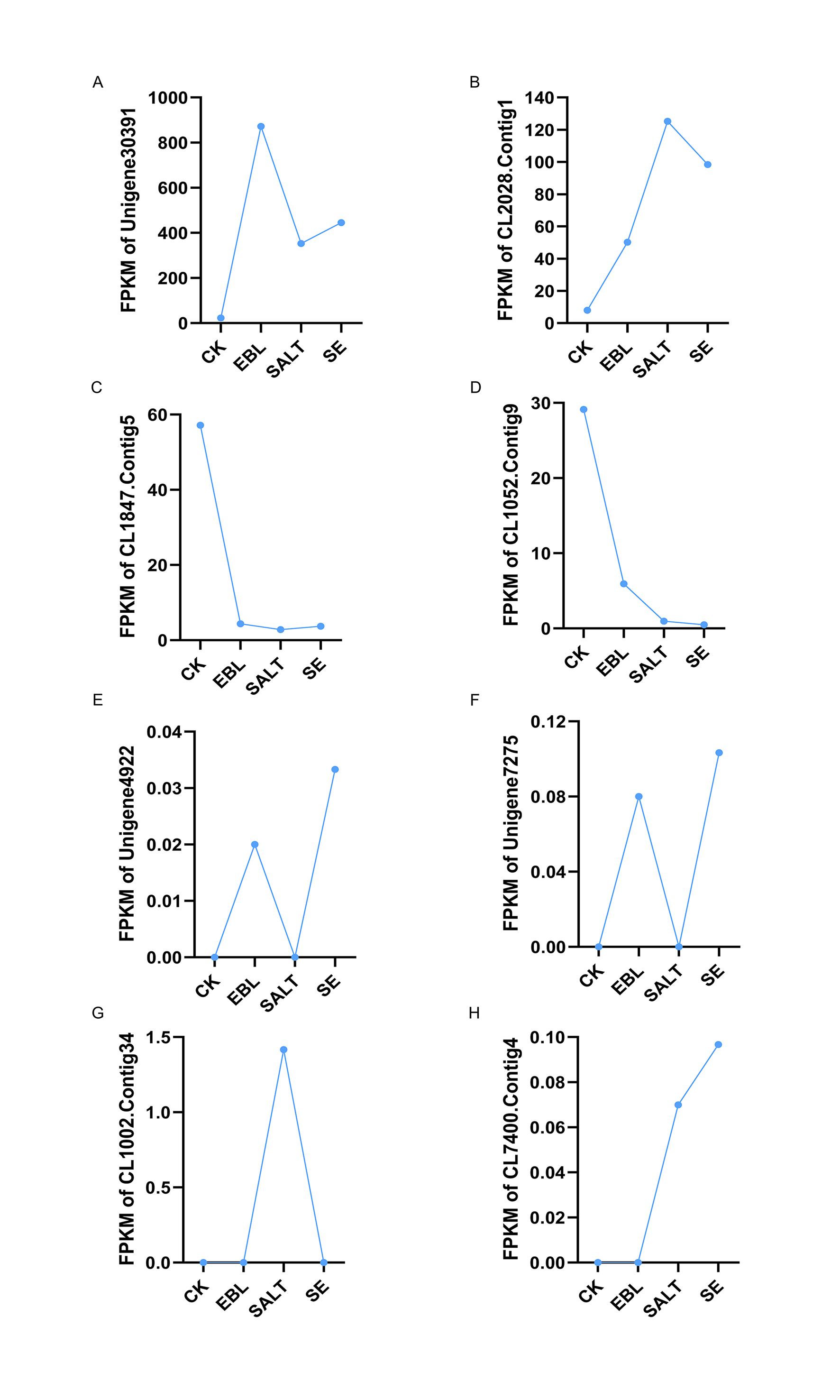

Supplement: Supplementary Figure 4 — FPKM values of genes used for qRT-PCR analysis. (A) FPKM values of Unigene30391; (B) FPKM values of CL2028.Contig1; (C) FPKM values of CL1847.Contig5; (D) FPKM values of CL1052.Contig9; (E) FPKM values of Unigene4922; (F) FPKM values of Unigene7275; (G) FPKM values of CL1002.Contig34; (H) FPKM values of CL7400.Contig4. [file Image_4.TIF]
